# The effect of diabetes and the common diabetogenic *TBC1D4* p.Arg684Ter variant on cardiovascular risk in Inuit in Greenland

**DOI:** 10.1038/s41598-020-79132-1

**Published:** 2020-12-16

**Authors:** Maria Overvad, Lars Jorge Diaz, Peter Bjerregaard, Michael Lynge Pedersen, Christina Viskum Lytken Larsen, Ninna Senftleber, Niels Grarup, Torben Hansen, Marit Eika Jørgensen

**Affiliations:** 1 National Institute of Public Health, University of Southern Denmark, Odense, Denmark; 2 Steno Diabetes Center Copenhagen, Gentofte, Denmark; 3 Greenland Center for Health Research, University of Greenland, Nuuk, Greenland; 4 Queen Ingrid Primary Health Care Center, Nuuk, Greenland; 5 The Bioinformatics Centre, Department of Biology, University of Copenhagen, Copenhagen, Denmark; 6 Novo Nordisk Foundation Center for Basic Metabolic Research, Faculty of Health and Medical Sciences, University of Copenhagen, Copenhagen, Denmark

**Keywords:** Cardiovascular diseases, Diabetes, Diabetes complications, Genetic predisposition to disease

## Abstract

Cardiovascular disease (CVD) is a well-known complication of diabetes, but the association has not been studied among Inuit in Greenland. The aim was to examine the association between diabetes and incident CVD among Inuit in Greenland and determine if the common diabetogenic *TBC1D4* variant confers increased risk of CVD. We followed an initial study population of 4127 adults in Greenland who had participated in at least one population-based health survey, in national registers. We used Poisson regression to calculate incidence rate ratios (IRR) of cardiovascular endpoints, comparing participants with and without diabetes and comparing homozygous *TBC1D4* carriers with heterozygous carriers and non-carriers combined. Close to 10% had diabetes and age range was 18–96 years (45% male). Of the 3924 participants without prior CVD, 362 (~ 9%) had CVD events during a median follow-up of 10 years. Multivariate IRR for the effect of diabetes on CVD was 1.12 (95% CI: 0.80, 1.57) *p* = 0.50. Using a recessive genetic model, we compared homozygous *TBC1D4* carriers with wildtype and heterozygous carriers combined, with a multivariate IRR of 1.20 (95% CI: 0.69, 2.11) *p* = 0.52. Neither diabetes nor the *TBC1D4* variant significantly increased CVD risk among Inuit in Greenland in adjusted models.

## Introduction

Cardiovascular disease (CVD) and diabetes are serious and costly conditions, associated with high morbidity and mortality. CVD is the number one cause of the death worldwide, accounting for roughly one third of all deaths^1^. Diabetes prevalence globally has almost doubled from 1980 to 2014 with a rise from 4.7% to 8.5%^2^. The same trend is seen in Greenland, where diabetes was almost non-existent half a century ago with a prevalence estimate of 0.06% in 1962^3^ in contrast to recent estimates of diabetes of 9% and prediabetes of 19%^4^. Diabetes increases risk of CVD two to three fold in European populations^5^ and the same trend is shown in Inuit populations^6,7^ including Inuit in Greenland^8,9^. However, no epidemiological studies have been conducted to this day, elucidating the effect of diabetes on CVD risk in Greenland.

Physical inactivity, obesity, ageing, poor diet, smoking, abnormal lipids, hypertension and genetic predisposition are common risk factors for diabetes^10–12^ and CVD^13–17^. More than half of the population in Greenland smoke^18^ and physical inactivity is much more common in modern days as a result of a social transition from a traditional active lifestyle to a modern sedentary life with access to processed high caloric foods^19^. Accordingly obesity is an increasing problem among Inuit in Greenland with a rise from 15 to 25% over the past two centuries^20^ and it is a major risk factor of diabetes^21^. The common myth that Inuit are exempted from CVD^22–24^ is continuously disproven with recent studies finding that CVDs in Inuit populations are prevalent and relevant public health issues^25–27^. Adding to the pool of risk factors for diabetes among Inuit is the unique genetic structure of isolated populations giving rise to common risk alleles with larger effect sizes than in populations with a more diverse genetic composition.

The *TBC1D4* p.Arg684Ter is one such risk variant discovered in the Greenlandic population in 2014 with an allele frequency of 17% and 4% homozygous (HO) carriers^28^. The diabetogenic effects demonstrated include decreased insulin-stimulated glucose uptake in skeletal muscle leading to postprandial hyperglycemia and hyperinsulinemia, impaired glucose tolerance and diabetes^28^. The variant shows the strongest association using a recessive model, and HO carriers have an odds ratio of 10.3 for developing diabetes and the variant is thought to explain around 15% of all diabetes in Greenland^28^. Among Inuit in Nunavik, a population comparable to Inuit in Greenland, diabetes was found to be underdiagnosed among carriers of the variant^29^, underlining the importance of recognizing potential clinical implications associated with the *TBC1D4* variant.

The pathophysiology connecting the *TBC1D4* variant to diabetes involves the defective protein it produces. The long isoform of *TBC1D4* expressed in skeletal and heart muscle codes for an insulin stimulated activating protein that facilitates GLUT4 translocation from intracellular vesicles to the cell membrane after insulin stimulation^30^. Lack of the long *TBC1D4* isoform confers postprandial hyperglycemia and hyperinsulinemia. A potential link between *TBC1D4* and CVD is supported by mice knock-out studies that have uncovered that TBC1D4 phosphorylation plays an important role in the electrical conduction system of the heart^31^ suggesting that the variant may influence cardiovascular outcomes. Furthermore, a recent *TBC1D4* study found that knock-out mice exhibited impaired cardiac function, increased infarction area and decreased left ventricular wall thickness three weeks after induced cardiac ischemia, compared to wild type (WT), indicating a functional role of *TBC1D4* variant regarding heart damage after myocardial infarction^32^. A common ground between the *TBC1D4* variant, diabetes and cardiovascular outcomes, is found in the “common soil hypothesis”, a shared pathophysiological pathway of diabetes and CVD through obesity, insulin resistance, dyslipidemia and inflammation^33,34^.

We do not know if CVD risk for Inuit with diabetes is the same as for Caucasians with diabetes or if the *TBC1D4* variant confers increased risk of CVD. The aim of the study was therefore to determine if diabetes is associated with CVD risk among Inuit in Greenland. The second objective was to examine if carriers of the *TBC1D4* variant have a higher risk of CVD than non-carriers.

## Methods

### Study population

The study population included participants from two countrywide Greenlandic population-based studies: the B99 study and the Inuit Health in Transition (IHIT) study. B99 is a population-based survey of life style and disease among Greenlanders conducted from 1999–2001 and IHIT, completed from 2005–2010, is a general health study among adults in Greenland^35,36^. Participants over 18 years who completed an interview, a clinical examination and had blood samples drawn, were included in the study population. Participants who appeared in both surveys were included at the earliest participation date to get maximum follow up time. The population surveys are described in detail elsewhere^35,36^. The studies were approved by the Ethics Committee in Greenland with adherence to the Declaration of Helsinki and written informed consent was obtained from participants.

### Definition of diabetes

Diabetes was defined according to World Health Organization 2006 criteria for type 2 diabetes: fasting plasma glucose > 6.9 mmol/l or plasma glucose > 11 mmol/l 2 h after an oral glucose load during an Oral Glucose Tolerance Test (OGTT) and/or self reported by questionnaire^37^.

### *TBC1D4* genotyping

Participants were genotyped for the *TBC1D4* variant using the KasPAR assay (LGC Genomics, Hoddesdon, UK) and admixture proportions of European ancestral DNA were estimated for each individual, where a proportion of 1 indicates 100% Inuit ancestry and zero indicates 100% European ancestry using data generated from the Illumina MetaboChip^28^.

### Cardiovascular outcomes

CVD outcomes were defined as fatal or non-fatal CVD diagnoses using the International Classification of Diseases 8 (ICD8) used from 1971–1993^38^, the International Classification of Diseases 10 (ICD10) used since 1994^39^, the International Classification of Primary Health Care 2 (ICPC2) used since 2003^40^ and the Danish version of the NOMESCO Classification of Surgical Procedures (SKS)^41^. We included five CVD groups namely ischemic heart disease (including coronary surgeries), stroke, vascular disease (including ischemic amputations), heart failure and atrial fibrillation. CVD outcomes were retrieved from the Greenlandic and Danish Hospital Discharge Registers and the Greenlandic and Danish Causes of Death Registers. CVD outcomes from national registers were linked to health surveys by the Personal Identification Number unique for every Greenlandic and Danish citizen (See Appendix).

### National registers

#### The Greenlandic Civil Registration System

The Greenlandic Civil Registration System is administered by the Danish Civil Registration System^42^. Amongst other things, it contains information on sex, place of birth, place of residence and emigration status.

#### The Greenlandic Hospital Discharge Register

Since 1987, the Greenlandic Hospital Discharge Register has been used to register discharge diagnoses from health centers and hospitals in Greenland. Medical information and information on surgical procedures are recorded. In 2014 Greenland changed to a new electronic medical record system from Æskulap to Cosmic. Cosmic was first implemented at Queen Ingrid’s Hospital in Nuuk in the beginning of 2014 and then successively among the 4 main regional hospitals and health centers throughout Greenland.

#### The Danish National Hospital Register

The Danish National Hospital Register was established in 1977 with the purpose of registering discharge diagnoses from Danish hospitals^43^. Greenlandic patients requiring specialized medical procedures or treatments unavailable in Greenland are flown to Copenhagen for treatment and their diagnoses are therefore found in this register.

#### The Danish Register of Causes of Death

Since 1875, deaths in Denmark have been registered by the National Board of Health and the Danish Register of Causes of Death was established in 1970 as a computerized record system^44^. The register includes, amongst other things, information on sex, age, causes of death and date of death for residents in Denmark dying in Denmark and Greenlandic residents dying in Denmark.

#### The Greenlandic Register of Causes of Death

The Greenlandic Register of Causes of Death was established in 1983 as part of the Danish Register of Causes of Deaths^44^. It includes causes of death of Greenlanders with a registered address in Greenland, as well as information on sex, age and date of death.

### Statistical analyses

We examined the association of diabetes with CVD risk and the effect of the *TBC1D4* p.Arg684Ter variant on CVD risk. Date of entry into the study was defined as date of the clinical examination during the health survey and participants were followed from entry until first CVD event, death, emigration or end of follow-up, whichever came first. CVD incidence rates were estimated using Poisson regression with log-person-time as offset variable and a significance level at 5%. Since CVD rates are not constant over time, the LEXIS macro was used to cut follow-up time into 1-year age bands and current age and calendar year were used as time scales^45^. Poisson regression was run on complete cases and participants with CVD events prior to entry were excluded from analyses. The effect of diabetes on CVD risk was estimated in a crude analysis and then adjusting for age, calendar year and sex in model 1. In model 2 we additionally adjusted for body mass index (BMI), systolic and diastolic blood pressure, low density lipoprotein (LDL) cholesterol, triacylglycerol (TAG) levels and smoking. In model 3 we further adjusted for the effect of *TBC1D4* variant and admixture. The effect of *TBC1D4* was estimated using a recessive model (the model with the best fit^28^) comparing HO with wildtype (WT) and heterozygous (HT) combined, in a crude model and adjusting for the same confounders as in model 3. As CVD outcomes related to diabetes are not consistently defined in the literature, we did a sensitivity analysis excluding atrial fibrillation and heart failure and ran analyses again (CVD outcomes then only included ischemic heart disease, stroke and vascular disease).

To avoid exclusion of individuals with missing data on OGTT and *TBC1D4* genotyping covariates, which may infer biased results^46^, we ran Multivariate Imputation by Chained Equation^47^ with missing-at-random assumptions. For both OGTT and *TBC1D4* missing data, we independently assessed five copies of the data, each with imputed missing values and estimates of parameters were averaged across the copies according to Rubin rules^48^. For imputation, we used R statistical program version 3.6.0 and the R-package mice^49^. Data management and Poisson regression was done using SAS Statistical Software (version 9.4)^50^.

## Results

The baseline study population included 4127 participants who had completed lifestyle questionnaires and clinical examinations in the two health surveys B99 (n = 1317) and IHIT (n = 2810). Age range was 18 to 96 years and 1837 (44%) were male. We found 367 (~ 10%) participants with diabetes out of 3653 who had complete OGTT information (Table 1) and as such 474 participants had missing data on fasting and/or 120 min blood glucose values of the OGTT. Participants with diabetes had higher median age, BMI, systolic and diastolic blood pressure and fewer were smokers than in the normoglycemia group. HO carriers of the *TBC1D4* variant were in higher number in the diabetes group compared to the normoglycemia group, n = 47 (13.7%) and n = 71 (2.3%) and conversely *TBC1D4* WT genotype was lower in the diabetes group than the normoglycemia group n = 205 (59.8%) and n = 2205 (70.3%) respectively.

**Table 1:** Baseline characteristics.

	N complete cases	Total (n = 4127)	Normoglycemia^a^ (n = 3286)	Diabetes^b^ (n = 367)
Males (%)	1837	1837 (44.5)	1449 (44.1)	176 (48.0)
Age	4127	43.8 [33.5, 54.6]	44.3 [35.9, 53.9]	57.3 [48.8, 66.6]
BMI (kg/m^2^)	4087	25.4 [22.5, 29.3]	25.4 [22.5, 29.2]	28.2 [23.3, 32.7]
Systolic blood pressure (mmHg)	4084	123.0 [112.0, 137.0]	123.5 [113.0, 136.5]	136.5 [122.5, 154.0]
Diastolic blood pressure (mmHg)	4084	76.0 [68.5, 84.0]	76.0 [68.5, 84.0]	80.0 [72.0, 88.5]
LDL cholesterol (mmol/l)	4092	3.5 [2.9, 4.3]	3.6 [2.9, 4.4]	3.6 [2.9, 4.4]
TAG (mmol/l)	4122	1.0 [0.8, 1.4]	1.0 [0.8, 1.4]	1.2 [0.8, 1.8]
Fasting glucose (mmol/l)	3968	5.6 [5.2, 6.0]	5.6 [5.2, 5.9]	7.2 [6.3, 7.9]
120 min glucose (mmol/l)	3623	5.4 [4.3, 6.8]	5.3 [4.2, 6.4]	9.9 [6.5, 13.5]
Smoking (%)	4035	2761 (68.4)	2246 (69.4)	194 (54.8)
*TBC1D4* HO (%)	3898	142 (3.6)	71 (2.3)	47 (13.7)
*TBC1D4* HT (%)	3898	1060 (27.2)	861 (27.5)	91 (26.5)
*TBC1D4* WT (%)	3898	2696 (69.2)	2205 (70.3)	205 (59.8)
Admixture proportion	4000	0.76 [0.61, 0.90]	0.76 [0.61, 0.91]	0.80 [0.64, 0.95]

Data are median [interquartile range] and n (%). ^a^474 participants with missing OGTT data excluded. ^b^Diabetes is both screen-detected and self-reported.

### Cardiovascular outcomes

We followed the initial study population of 4127 individuals in national registers for a median of 10.2 years interquartile range (IQR) [8.3, 13.8] until CVD event, death, emigration or end of follow-up (December 31st 2016) whichever came first. A total of 622 participants died during follow-up and 111 were CVD registered deaths. We excluded 203 individuals who had a CVD event registered prior to inclusion and of the 3924 individuals that remained 362 (9.2%) had incident CVD events (Table 2). We divided participants with complete OGTT data n = 3460 (464 with missing OGTT data) into diabetes (n = 319) and normoglycemia (n = 3141) groups that accounted for 60 (18.8%) and 283 (9.0%) CVD events respectively. In total, CVD outcomes were most commonly stroke (35.1%) and ischemic heart disease (34.8%) and to a lesser extent heart failure (13.8%), atrial fibrillation (13.3%) and vascular disease (3.0%). In the diabetes group compared to the normoglycemia group, we found a higher frequency of diagnoses of heart failure (16.7% vs 13.1%), atrial fibrillation (16.7% vs 13.1%) and CVD deaths (33.3% vs 29.7%) and fewer diagnoses of stroke (30.0% vs 36.4) and vascular disease (1.7% vs 3.5%).

**Table 2:** Cardiovascular outcomes.

	Total^a^ (n = 3924)	Normoglycemia^b^ (n = 3141)	Diabetes^c^ (n = 319)
CVD events including fatal	362	283	60
Ischemic heart disease	126 (34.8)	96 (33.9)	21 (35.0)
Heart failure	50 (13.8)	37 (13.1)	10 (16.7)
Stroke	127 (35.1)	103 (36.4)	18 (30.0)
Vascular disease	11 (3.0)	10 (3.5)	1 (1.7)
Atrial fibrillation	48 (13.3)	37 (13.1)	10 (16.7)
CVD deaths	111 (30.7)	84 (29.7)	20 (33.3)

Data are n and (%) of “CVD events including fatal”. ^a^203 with prior CVD events excluded. ^b^464 participants missing OGTT data excluded. ^c^Diabetes is both screen-detected and self-reported.

### *TBC1D4* genotype distribution among CVD events

Of the 3924 participants we included without previous CVD events 3705 were genotyped for the *TBC1D4* variant and 330 of the genotyped participants developed CVD events (Table 3). *TBC1D4* genotypes of those who had CVD events were 217 (65.8%) WT, 98 (29.7%) HT and 15 (4.5%) HO. This corresponds to 8.5% of WT, 9.7% of HT and 11.4% of HO genotypes developing CVD events. Types of CVD events seemed equally distributed among genotypes, except that HO genotype had fewer strokes (20% vs 35.5%) and more ischemic heart disease (46.7% vs 32.7%) compared to WT genotype. CVD deaths were also more frequent among HO than WT (33.3% vs 27.2).

**Table 3:** *TBC1D4* genotype distribution among CVD events.

	Total genotyped^a^ (n = 3705)	Homozygous (n = 132)	Heterozygous (n = 1013)	Wildtype (n = 2560)
CVD events including fatal	330	15	98	217
Ischemic heart disease	112 (34.0)	7 (46.7)	34 (34.7)	71 (32.7)
Heart failure	46 (13.9)	2 (13.3)	12 (12.2)	32 (14.8)
Stroke	119 (36.1)	3 (20.0)	39 (39.8)	77 (35.5)
Vascular disease	8 (2.4)	1 (6.7)	1 (1.0)	6 (2.8)
Atrial fibrillation	45 (13.6)	2 (13.3)	12 (12.2)	31 (14.3)
CVD deaths	99 (30.0)	5 (33.3)	35 (35.7)	59 (27.2)

Data are n and (%) of “CVD events including fatal”. ^a^ 219 participants missing genotype information excluded.

### The effect of diabetes on CVD risk and all-cause mortality

The effect of diabetes on CVD risk estimated by Poisson regression is shown in Table 4. We found significantly increased risk of CVD for participants with diabetes with a crude incidence rate ratio (IRR) of 2.45 (95% CI: 1.85, 3.23) *p* < 0.0001. However, in model 1 (adjusted for age, sex and calendar year) IRR decreased to 1.32 (95% CI: 0.99, 1.75) *p* = 0.061 and in model 2 (further adjusting for BMI, systolic and diastolic blood pressure, LDL cholesterol, TAG and smoking) IRR decreased to 1.16 (95% CI: 0.85, 1.59) *p* = 0.34. In model 3 (further adjusting for *TBC1D4* and admixture) IRR was 1.12 (95% CI: 0.80, 1.57) *p* = 0.50. We did multiple imputation on missing OGTT data (n = 464 with 19 CVD events) and new Poisson regression on imputed datasets did not change IRR significantly (data not shown) suggesting that participants with missing OGTT data are not much different from complete cases. In model 3 with all-cause mortality as outcome, the effect of diabetes increased risk of death by an IRR of 1.32 (11.04,1.67) *p* = 0.0.

**Table 4:** The effect of diabetes and a recessive model of the *TBC1D4* variant on CVD risk.

	N complete cases	Crude IRR for CVD	Model 1 IRR for CVD	Model 2 IRR for CVD	Model 3 IRR for CVD
Diabetes ^a^	3460	**2.45** (1.85, 3.23) *p* < 0.0001*	1.32 (0.99, 1.75) *p* = 0.061	1.16 (0.85, 1.59) *p* = 0.34	1.12 (0.80, 1.57) *p* = 0.50
Age	3460		**1.07** (1.06, 1.00) *p* < 0.0001*	**1.07** (1.06, 1.08) *p* < 0.0001*	**1.07** (1.06, 1.08) *p* < 0.0001*
Sex (male vs female)	3460		**1.52** (1.23, 1.87) *p* = 0.0001*	**1.48** (1.18, 1.85) *p* = 0.0007*	**1.45** (1.15, 1.83) *p* < 0.0001*
Calendar year	3460		**1.03** (1.00, 1.05) *p* = 0.034*	1.03 (1.00, 1.05) *p* = 0.07	1.02 (0.99, 1.05) *p* = 0.15
BMI (kg/m^2^)	3338			1.00 (0.97, 1.02) *p* = 0.68	1.01 (0.98, 1.03) *p* = 0.69
Systolic BP (mm/Hg)	3338			1.01 (1.00, 1.01) *p* = 0.13	1.01 (1.00, 1.01) *p* = 0.17
Diastolic BP (mm/Hg)	3338			1.01 (0.99, 1.01) *p* = 0.77	1.00 (1.00, 1.14) *p* = 0.69
LDL (mmol/l)	3338			**1.17** (1.06, 1.30) *p* = 0.002*	**1.16** (1.05, 1.29) *p* = 0.005*
TAG (mmol/l)	3338			1.10 (0.91, 1.32) *p* = 0.32	1.10 (0.91, 1.33) *p* = 0.32
Smoking	3338			1.09 (0.85, 1.39) *p* = 0.50	1.13 (0.87, 1.45) *p* = 0.36
*TBC1D4* HO vs WT + HT	3194				1.20 (0.69, 2.11) *p* = 0.52
Admixture proportion	3194				0.66 (0.37, 1.17) *p* = 0.16

Poisson regression with incidence rate ratios (IRR) and (confidence limits). *Effect sizes in bold below 5% significance level. ^a^Diabetes is both screen-detected and self-reported. Model 1: adjusted for age, sex and calendar year; Model 2: model 1 + BMI, systolic BP, diastolic BP, LDL cholesterol, TAG and smoking; Model 3: model 2 + *TBC1D4* and admixture.

### The effect of the *TBC1D4* variant on CVD risk and all-cause mortality

Assuming a recessive effect of the *TBC1D4* variant on CVD risk we found a crude IRR of 1.37 (95% CI: 0.81, 2.29) *p* = 0.24 for HO vs WT + HT. In model 3 (Table 4), we adjusted for admixture in addition to all previous covariates and IRR was 1.20 (95% CI: 0.69, 2.11) *p* = 0.52. We found an Inuit protective but statistically insignificant effect of admixture on CVD risk, with IRR of 0.66 (95% CI: 0.37, 1.17) *p* = 0.15 per percentage increase in Inuit admixture. In order to address missing data on genotypes (n = 219 with 32 CVD events) we did multiple imputation. In both crude models and model 3 run again with imputed data, IRR remained almost unchanged (data not shown), suggesting that participants with missing genotype data are not markedly different from complete cases. In model 3 with all-cause mortality as outcome, the effect of HO carrier status vs non-HO was an IRR of 0.84 (0.52, 1.35) *p* = 0.47.

### Sensitivity analysis of CVD outcomes

In a sensitivity analysis of CVD outcomes, we omitted atrial fibrillation and heart failure and kept stroke, ischemic heart disease and vascular disease. With fewer CVD outcomes effect sizes became lower with IRR of 2.18 (95% CI: 1.61, 2.96) *p* < 0.0001 in a crude analysis and in model 1 IRR was 1.21 (95% CI: 0.89, 1.65) *p* = 0.23 (age, sex and calendar year significantly associated with increasing CVD risk, data not shown). In model 2 IRR was 1.12 (95% CI: 0.80, 1.56) *p* = 0.51 (age, sex, calendar year and LDL significantly associated with increased risk, data not shown). In model 3 using the recessive model IRR for HO vs WT + HT was 1.07 (95% CI: 0.74, 1.54) *p* = 0.72 (age, sex, calendar year, LDL cholesterol, smoking and European admixture increased CVD risk significantly, data not shown).

## Discussion

In this study we tested if diabetes and the *TBC1D4* variant were associated with CVD risk in Inuit in Greenland. We found that diabetes was associated with elevated CVD risk in a crude analysis, however, in adjusted models the effect was not maintained. Assuming a recessive effect the *TBC1D4* variant showed a maintained trend of increased CVD risk, however this trend was not significant and a protective effect on all-cause mortality was not significant either. Age, male sex and LDL cholesterol significantly increased CVD risk in adjusted models as has been described in Inuit populations before^51^. In total 9% of normoglycemic participants and 19% of participants with diabetes developed incident CVD events. Altogether ~ 10% of participants had diabetes, as has been described before in the Greenlandic population^52^. Diabetes conferred a significant increase of all-cause mortality in a multi-adjusted model. It was unexpected that diabetes did not confer statistically significant increased CVD risk, as is the case in many other non-Inuit populations^5,53–55^ and in Inuit in Alaska^51^. We propose several reasons to explaining this.

One reason could be if CVD outcomes were not defined accurately. The literature is rich in studies supporting that diabetes increases CVD risk, but definition of CVD outcomes varies greatly. Agreeing upon which CVD diagnoses are diabetes relevant is difficult and especially atrial fibrillation and heart failure may cause discussions among physicians. We chose to include atrial fibrillation^23,56,57^ and heart failure^58–60^, supported by the literature as relevant complications to diabetes, in addition to stroke, ischemic heart disease and vascular disease. In a sensitivity analysis we omitted atrial fibrillation and heart failure. Age, male sex and LDL remained significant and in addition we found that smoking, calendar year and European admixture significantly increased risk of CVD. The effect of smoking could be explained by its pathophysiological mechanisms causing atherosclerosis, which is a dominant factor in the pathophysiology of especially ischemic heart disease, vascular disease and stroke^51^. Atrial fibrillation and heart failure have multiple other pathophysiological mechanisms besides atherosclerosis harmonizing well with the effect of smoking found when these were omitted. The elevated CVD risk per increase in calendar year may walk hand in hand with the traditional Inuit diet of marine animals steadily over time being replaced by imported foods of low quality, thus increasing CVD associated risk^62^. One study of Inuit in Alaska found that a traditional Inuit diet was associated with lower TAG, blood pressure and slightly higher LDL, concluding that it was linked to a better cardiovascular profile^63^. The fact that Inuit admixture had a protective effect on CVD risk in the sensitivity analysis, could also be related to the Inuit diet being more habitual among inhabitants of smaller more isolated villages in Greenland, where Inuit admixture levels are highest^64^.

A second reason could be that Inuit are not directly comparable to Caucasians due to genetic differences. Genes play a powerful role in isolated populations as seen among Pima Indians where close to half of the population is diagnosed with diabetes^65,66^. Similarly, the *TBC1D4* variant convincingly increases diabetes risk in Inuit populations, but it does not seem to convincingly increase CVD risk. An explanation could be, that there could be undiscovered CVD protective variants or Inuit specific interactions with diet or metabolic traits affecting CVD risk, or the *TBC1D4* variant could induce less dangerous diabetes form like GCK-MODY (Glycokinase Maturity Onset Diabetes of the Young). GCK-MODY patients have prolonged mild hyperglycemia but do not require treatment^67^. The *TBC1D4* variant showed a protective but not statistically significant effect on all-cause mortality and diabetes significantly increased risk of all-cause mortality, supporting a theory that *TBC1D4* diabetes may have fewer clinical implications than non-*TBC1D4* diabetes.

A third reason could be that diabetes duration was not long enough to confer increased CVD risk, as diabetes incidence in Greenland has increased from virtually non-existent to ~ 10% over the last half a century. Perhaps in 50 years, diabetes associated CVD risk will be comparable to Caucasian populations. CVD risk increases with age and although life expectancy in Greenland has gone up from 63 to 72 years, compared to the mean of the European Union of 72 to 82 years over the past 40 years^68^, it remains substantially lower and a younger population will develop fewer CVD events.

A fourth reason could be changes in health behavior after receiving a diabetes diagnosis. Participants with diabetes in this study were screen detected in population-based studies and were identified early in time, possibly before any diabetes symptoms manifested themselves. Consequently, participants identified with diabetes would have had time to change their risk profile by e.g. smoking secession, weight loss, a healthier diet and so on. We found fewer smokers in the diabetes group compared to the normoglycemia group 54.8 vs 69.4%. As such CVD risk may be underestimated.

A fifth explanation could be an imprecise definition of diabetes in this population. We used self-reported and OGTT (fasting and two-hour glucose measurements) to define diabetes, as WHO guidelines suggested at the time the health surveys were conducted. Using glycated hemoglobin (Hba1c) we would have caught an overlapping but not identical group of people with diabetes. A study with Inuit in Canada with the same allele frequency and postprandial effect of *TBC1D4*, found that in carriers with prediabetes or type 2 diabetes, 32% would have remained undiagnosed without an OGTT^29^. Defining diabetes by Hba1c instead of OGTT criteria as been shown to affect ethnic groups differently^69^. Inuit have, for any given glucose value by OGTT, a higher Hba1c compared to Danes, suggesting different associations between Hba1c and blood glucose levels in these populations^70^.

A sixth reason could be if the registers with CVD outcomes were incomplete or faulty. However this seems unlikely, because two studies validated the Greenlandic Hospital Discharge Register in its entirety^71^ and CVD diagnoses separately^72^ and both found it valid for epidemiological purposes. Diagnoses in the Danish Hospital Discharge Register have also been validated with good results^43^.

Regarding the effect of the *TBC1D4* variant*,* we found maintained increased but statistically insignificant risk of CVD for HO carriers. We handled missing genotypes by redoing Poisson regression after multiple imputation and estimates were not changed significantly, suggesting that the number of complete cases in the models are representative of the full sample.

Compared to non-carriers, HO carriers had more frequent ischemic heart disease (HO 46.7% vs WT 32.7%), CVD deaths (33.3% vs 27.2) and fewer stroke diagnoses (HO 20.0% vs WT 35.5%), corresponding well to the fact that *TBC1D4* is expressed in heart and muscle tissue and not in the brain. Although we could not detect a significantly increased risk of CVD for the *TBC1D4* variant, we cannot completely rule one out. Since the *TBC1D4* variant is present with the same allele frequency among Inuit in Canada^29^, a way to get more power could be to pool data from Greenland and Canada. If there is an increased risk of CVD for HO carriers of the *TBC1D4* variant, it would be of public health interest to uncover it.

## Conclusions

In conclusion, this study showed that neither diabetes nor the *TBC1D4* variant significantly increased CVD risk in Inuit in Greenland. However, we find it imprudent to draw hasty conclusions and suggest more studies be conducted on diabetes and CVD risk in Inuit populations.

### Ethics approval and consent to participate

Oral and written consent was obtained from all participants in the two health surveys (B99 and IHIT) and the committee for Research Ethics in Greenland (Commission for Scientific Research in Greenland) granted ethical approval. The study protocols were in accordance with the Helsinki Declaration.

## Supplementary information

Supplementary Information 1.

## Data Availability

Data of the health surveys is available upon request for relevant purposes.

## Abbreviations

CI: Confidence interval
CVD: Cardiovascular disease
HO: Homozygous
HT: Heterozygous
IRR: Incidence rate ratio
LDL: Low density lipoprotein
OGTT: Oral glucose tolerance test
TAG: Triacylglycerol
WT: Wildtype
