# Supplementary material for: The effect of diabetes and the common diabetogenic TBC1D4 p.Arg684Ter variant on cardiovascular risk in Inuit in Greenland
Source: Sci Rep. 2020 Dec 16;10:22081. doi: 10.1038/s41598-020-79132-1 (PMC7745023; doi:10.1038/s41598-020-79132-1)
Supplement: Supplementary file 1 — Supplementary Information 1. [file 41598_2020_79132_MOESM1_ESM.docx]

**The effect of diabetes and the common diabetogenic *TBC1D4* p.Arg684Ter variant on cardiovascular risk in Inuit in Greenland**

*Maria Overvad^,2^, Lars Jorge Diaz ^2^, Peter Bjerregaard^1^, Michael Lynge Pedersen^3,4^, Christina Viskum Lytken Larsen^1,3^, Ninna Senftleber^2,5^, Niels Grarup^6^, Torben Hansen^6^, Marit Eika Jørgensen ^1,2,3^*

^1^ National Institute of Public Health, University of Southern Denmark, Denmark; ^2^ Steno Diabetes Center Copenhagen, Gentofte, Denmark; ^3^ Greenland Center for Health Research, University of Greenland, Nuuk, Greenland; ^4^ Queen Ingrid Primary Health Care Center, Nuuk, Greenland; ^5^ The Bioinformatics Centre, Department of Biology, University of Copenhagen, Copenhagen, Denmark.^6^ Novo Nordisk Foundation Center for Basic Metabolic Research, Faculty of Health and Medical Sciences, University of Copenhagen, Copenhagen, Denmark.

**Appendix 1: Cardiovascular diagnoses codes included. ICD8 and SKS, ICD10 and SKS and ICPC2 diagnoses**

| **Group** | **ICD8** | **SKS ICD8** | **ICD10** | **SKS ICD10** | **ICPC2** |
| --- | --- | --- | --- | --- | --- |
| **Ischemic heart disease**  **(including SKS coronary bypasses)** | 41009 41099 41109 41199 41209 41299 41309 41399 41409 41499 | 30009 30019 30029 30039 30049 30059 30069 30079 30089 30099 30109 30119  30120 30129 30139 30149 30159 30169 30179 30189 30199 30200 30240 30241  30245 30350 30354 30359 | DI20 DI21 DI22 DI23 DI24 DI25 | KFNA KFNB  KFNC KFND  KFNE KFNF  KFNG KFNH  KFNW96 KFNW98 | K74  K75  K76 |
| **Stroke** | 43100 43101 43108 43109 43190 43191 43198 43199 43200 43201 43202 43208  43209 43290 43291 43292 43293 43298 43299 43309 43399 43409 43499 43509  43599 43601 43609 43690 43699 43700 43701 43708 43709 43790 43791 43798  43799 |  | DI60 DI61 DI62 DI63 DI64 DI65  DI66 DI672 DI678 DI693 DI694 |  | K90  K91 |
| **Vascular disease**  **(including SKS ischemic amputations)** | 44009 44019 44020 44021 44028 44029 44039 44099 44408 44409 44419 44420  44421 44428 44429 44439 44440 44441 44442 44443 44444 44448 44449 44490  44499 | 81031 81040 81041 81050 81051 81052  81053 81054 81055 81059 81060 81080  81081 81100 81190 | DI70 DI739 DI739A DI739C DI740 DI740B DI740D DI741 DI742 DI743  DI744 DI745  DI748 DI749 | KNGQ  KNHQ | K92  K99 |
| **Heart failure** | 42709 42710 42711  42719 42899 78249 |  | DI50 |  | K77 |
| **Atrial fibrillation** | 42793 42794 |  | DI48 DI480  DI481 DI482 DI483 DI484  DI489 |  | K78 |
